# Regulation of Vascular Function and Inflammation via Cross Talk of Reactive Oxygen and Nitrogen Species from Mitochondria or NADPH Oxidase—Implications for Diabetes Progression

**DOI:** 10.3390/ijms21103405

**Published:** 2020-05-12

**Authors:** Andreas Daiber, Sebastian Steven, Ksenija Vujacic-Mirski, Sanela Kalinovic, Matthias Oelze, Fabio Di Lisa, Thomas Münzel

**Affiliations:** 1 Center for Cardiology, Department of Cardiology 1, University Medical Center of the Johannes Gutenberg University, 55131 Mainz, Germany; sesteven@uni-mainz.de (S.S.); ksenija.vujacic.mirski@gmail.com (K.V.-M.); sanelakalinovic@gmail.com (S.K.); matzeoelze@aol.com (M.O.); 2 Partner site Rhine-Main, German Center for Cardiovascular Research (DZHK), 55131 Mainz, Germany; 3 Department of Biomedical Sciences, University of Padova, 35131 Padova, Italy; dilisa@bio.unipd.it

**Keywords:** redox cross talk, mitochondria, NADPH oxidase, kindling radicals, oxidative stress, endothelial dysfunction, eNOS uncoupling, low-grade inflammation

## Abstract

Oxidative stress plays a key role for the development of cardiovascular, metabolic, and neurodegenerative disease. This concept has been proven by using the approach of genetic deletion of reactive oxygen and nitrogen species (RONS) producing, pro-oxidant enzymes as well as by the overexpression of RONS detoxifying, antioxidant enzymes leading to an amelioration of the severity of diseases. Vice versa, the development and progression of cardiovascular diseases is aggravated by overexpression of RONS producing enzymes as well as deletion of RONS detoxifying enzymes. We have previously identified cross talk mechanisms between different sources of RONS, which can amplify the oxidative stress-mediated damage. Here, the pathways and potential mechanisms leading to this cross talk are analyzed in detail and highlighted by selected examples from the current literature and own data including hypoxia, angiotensin II (AT-II)-induced hypertension, nitrate tolerance, aging, and others. The general concept of redox-based activation of RONS sources via “kindling radicals” and enzyme-specific “redox switches” as well as the interaction with redox-sensitive inflammatory pathways are discussed. Here, we present evidence for the existence of such cross talk mechanisms in the setting of diabetes and critically assess their contribution to the severity of diabetic complications.

## 1. Introduction

### 1.1. Reactive Oxygen and Nitrogen Species in the Organism

A molecular proof of the existence of superoxide anion radical (O_2_^•^¯) formation in the organism was based on the discovery of superoxide dismutases (SODs, mitochondrial Mn-SOD, and cytosolic/extracellular Cu,Zn-SOD) in living organisms by Fridovich and coworkers in the 1960s [1]. The existence of SODs in biological systems also suggests that O_2_^•^¯is a harmful species involved in pathophysiological processes and that the expression of SODs is mandatory to protect the organism from oxidative damage by superoxide. Although the degradation product of O_2_^•^¯, hydrogen peroxide (H_2_O_2_), may confer redox signaling by oxidation of protein thiol groups and act as an important second messenger that is essentially involved in fundamental cellular processes such as eustress [2,3] or cell differentiation/proliferation [4], its concentrations need to be tightly controlled by catalase and glutathione peroxidases (GPx) to prevent oxidative stress conditions and exaggerated oxidative damage of cellular structures [5]. Other important physiological functions of hydrogen peroxide comprise the formation of disulfide bonds during “oxidative” protein folding in the endoplasmic reticulum [6,7] and providing the “peroxide tone” for enzymes such as cyclooxygenases [8]. Redox signals by hydrogen peroxide are either directly mediated by sulfoxidation and *S*-glutathionylation of thiol-dependent enzymes or via modulation of the oxidation state of thiols in peroxiredoxins, thioredoxins, and glutaredoxins that are all interconnected and coupled to their reductases using NAD(P)H as an electron-providing cofactor [9,10]. More examples on hydrogen peroxide-dependent redox signaling are provided in the position paper by the EU-ROS COST Action [11]. The beneficial effects of ROS may also represent the reason why most large-scale clinical trials on antioxidants failed or even turned-out negative [12], indicating that unspecific systemic inhibition of ROS formation may inhibit these physiological, beneficial functions of ROS and thereby promote adverse health effects.

In contrast, O_2_^•^¯ seems to be more harmful than H_2_O_2_ since genetic deficiency in Mn-SOD is lethal at the embryonic stage or shortly after birth due to heart failure and neurological disorders [13,14]. The only “real” physiological role of superoxide potentially comprises its role in host defense as *Nox2*^−/−^ mice [15] and patients with chronic granulomatous disease (*Nox* gene mutations) [16] are more susceptible to infections. The harmful or antibacterial properties of O_2_^•^¯ may be explained not only by the high reactivity of O_2_^•^¯ towards transition metal complexes (e.g., iron–sulfur clusters in mitochondrial proteins of the respiratory chain or the central phosphatase calcineurin) but also by its fast reaction with nitric oxide (^•^NO) [17,18]. After two decades of intensive research (1970s and 1980s) ^•^NO was identified as the ”endothelium-derived relaxing factor“ (EDRF), a potent vasodilator by its activation of soluble guanylyl cyclase (sGC) in the smooth muscle, which was a joint effort by the Noble Prize recipients Murad, Ignarro, und Furchgott [19,20,21]. This discovery changed the negative picture that scientists had of free radicals in biology and helped to understand that these species can also confer cellular redox signaling and thereby act as highly important physiological messenger molecules. The physiological role of ^•^NO as a vasodilator and as a neurotransmitter was extensively reviewed [22,23,24,25]. In the 1990s, it became evident that O_2_^•^¯ reacts with ^•^NO with almost diffusion-controlled kinetics leading to the formation of peroxynitrite (ONOO¯) [26], which leaves its footprints in vivo by nitration of protein-bound tyrosine residues [27,28,29] that can be detected by specific antibodies against 3-nitrotyrosine-positive proteins, e.g., in atherosclerotic plaques [30,31,32]. The formation of hydroxyl radicals (HO^•^) is a driving force of the oxidative potential of ONOO¯ [33] and its nitrating potential is enhanced in the presence of carbon monoxide [34] or transition metal centers, e.g., of manganese, heme, or heme-thiolate (P450) enzymes [35,36,37,38,39,40].

**Figure 1:**
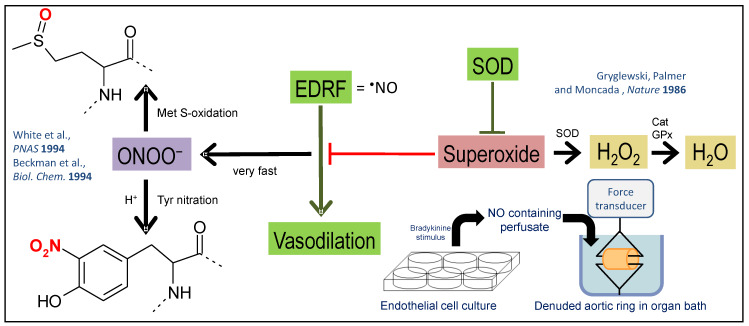
Overview on the simplified model of redox biology in the vascular system. O_2_^•^¯ was identified as an antagonist of the EDRF (see red inhibitory bar), far before EDRF was widely accepted to be ^•^NO by the famous experiment of Gryglewski, Palmer, and Moncada based on the transfer of the perfusate from bradykinin-stimulated endothelial cell culture to an organ bath with denuded (endothelium-devoid) aortic ring segments [44]. The vasodilatory potency of EDRF coming from the cell culture was increased by addition of SOD to the buffer on the cells conferring dismutation of O_2_^•^¯ (see green inhibitory bar), supporting the break-down of EDRF by O_2_^•^¯. From previous work, we know today that ^•^NO and O_2_^•^¯ react in a diffusion-controlled reaction to form ONOO¯ [30,31]. Without this reaction, O_2_^•^¯ is dismutated either by SODs or undergoes spontaneous self-dismutation to form H_2_O_2_, which is largely involved in redox signaling pathways via oxidation of specific thiol residues, or inactivated by catalases (Cat), GPx, or peroxiredoxins (Prx). ONOO¯ can cause widespread oxidative damage in proteins (tyrosine nitration [3-NT] and methionine sulfoxidation [oxMet]) but also lipids and DNA molecules [54]. Scheme is modified from [41] with permission.

**Figure 2:**
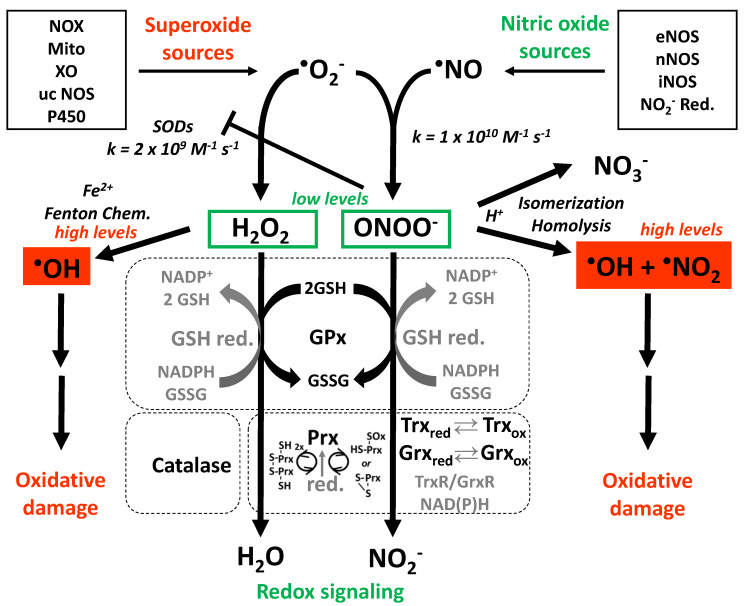
The major pathways of vascular oxidative stress and redox signaling. Redox signaling is mainly based on H_2_O_2_ that is formed by breakdown of O_2_^•^¯ via self-dismutation or catalyzed by SODs. Biological O_2_^•^¯ sources are NADPH oxidases (NOX), the mitochondrial respiratory chain (Mito), xanthine oxidase (XO), an uncoupled NOS (ucNOS), and P450 enzyme side reactions. H_2_O_2_ modulates the thiol/disulfide equilibrium and thereby modifies enzymatic activities (e.g., in zinc-finger-motifs as found in transcription factors). Reaction with thiol groups is also a major route of detoxification for H_2_O_2_ via peroxiredoxins (Prx), glutaredoxins (Grx), and thioredoxins (Trx) or the low-molecular-weight thiol glutathione (GSH) that may be coupled to the faster reacting selenol in GPx—these systems require energy-consuming recycling by NAD(P)H-coupled reductases, are highly interconnected, and form a complex redox network that also affects thiol groups in other enzymes [9,10]. Decomposition of H_2_O_2_ is also catalyzed by catalase. Accumulation of H_2_O_2_ leads to the Fenton reaction and hydroxyl radical (HO^•^) formation, which is based on the reaction of H_2_O_2_ with ferrous iron (Fe^2+^), yielding ferric iron (Fe^3+^) that is reduced back to ferrous form by O_2_^•^¯ (the sum of these reactions is called the Haber–Weiss cycle). Hydroxyl radicals cause severe oxidative damage at the protein, lipid, and DNA level. Biological ^•^NO sources are neuronal, endothelial, or inducible NOS as well as the reduction of nitrite from nutritional sources. The diffusion-controlled reaction of ^•^NO with O_2_^•^¯ yields ONOO¯ anion and is fast enough to even outcompete the extremely fast breakdown of O_2_^•^¯ by SOD. Kinetic considerations support the formation of ONOO¯ under physiological and, especially, under pathophysiological conditions. The reported inactivation of SOD isoforms (via nitration/dityrosine formation in SOD2 and damage of the Cu,Zn-complex in SOD1/3) lead to a further increase in O_2_^•^¯ levels in a positive-feedback fashion. The redox signaling mechanisms by ONOO¯ are similar to those mediated by H_2_O_2_, but ONOO¯ (or its conjugated acid) has 100–1000-fold higher reactivity. Once protonated, ONOOH can be degraded by spontaneous isomerization to nitrate or can be activated by homolysis to form the HO^•^ and the nitrogen dioxide (^•^NO_2_) radicals with a similar oxidative damage profile as observed for the Fenton reaction. Scheme is significantly modified from [55].

In many aspects, O_2_^•^¯ can be regarded as direct antagonist of ^•^NO [41,42,43], a concept that was already proven in 1986 by demonstrating that SOD prevents the loss of vasodilatory effects of ^•^NO, formerly known as EDRF, in denuded vessels (Figure 1) [44]. The oxidative degradation of ^•^NO by O_2_^•^¯ directly contributes to endothelial dysfunction by removal of a potent vasodilator. In addition, the formation of ONOO¯ causes oxidative damage of important vascular proteins, e.g., endothelial nitric oxide synthase (eNOS) [45,46], sGC [47], and prostacyclin synthase (PGIS) [48] and thereby contributes to endothelial (vascular) dysfunction [49,50]. Endothelial (vascular) dysfunction of the micro- and macrovascular system also represents a major health risk of diabetic patients [51,52,53]. The interplay and steady-state levels of O_2_^•^¯, ^•^NO, and their reaction product ONOO¯ as well as their tight control by antioxidant enzymes largely determine cellular redox state and whether RONS at low concentrations act as messengers in redox signaling or at high concentrations cause oxidative stress and damage of biomolecules (Figure 2) [11].

### 1.2. Sources of Reactive Oxygen and Nitrogen Species

O_2_^•^¯ can be formed from different sources such as xanthine oxidase (XO), NOX, ucNOS, and the mitochondrial respiratory chain as well as H_2_O_2_ by specific mitochondrial enzymes such as monoamine oxidase (MAO) or p66^Shc^/cytochrome c complex. As all of these O_2_^•^¯ sources were extensively reviewed in the past [56], their function and isoforms are only briefly summarized here. XO is a molybdenum/iron enzyme that transfers electrons from hypoxanthine or xanthine (also other substrates, e.g., acetaldehyde) to molecular oxygen to produce O_2_^•^¯. Two meta-analyses of clinical studies concluded that XO inhibition may be cardioprotective [57,58]. NOX exist in different isoforms NOX1-5 and dual oxidase (DUOX)1-2. They are multiprotein complexes with transmembrane-spanning domains. The catalytic heme subunit transfers electrons from NADPH to molecular oxygen to produce O_2_^•^¯. Most prominent isoforms are: NOX2, the phagocytic NOX, that is highly regulated by cytokines as well as AT-II via protein kinase C (PKC) and that is highly expressed in phagocytic cells (e.g., leukocytes and macrophages) and has a role in inflammation; NOX1 that is highly expressed in the vasculature and regulated comparable to NOX2; and NOX4, the constitutive NOX that produces low levels of H_2_O_2_ for maintenance of essential cellular functions. NOX represent the only source of O_2_^•^¯ that has no other biological function but to produce O_2_^•^¯. Therefore, NOX attracted pharmacological interest and provides the basis for therapeutic targeting [56,59], but so far, none of these compounds reached application in the clinics. NOSs exist in different isoforms NOS1-3 (neuronal, inducible, and endothelial NOS). They usually produce ^•^NO by conversion of the substrate L-arginine to L-citrulline by electron transfer from NADPH and reaction of molecular oxygen with the help of the heme-iron and the cofactor tetrahydrobiopterin (BH4). ucNOS isoforms produce O_2_^•^¯ instead of ^•^NO by electron transfer from NADPH to molecular oxygen, e.g., upon depletion of BH4 or in the absence of L-arginine [49,60]. There is no drug in clinical use for recoupling of NOS but therapeutic targeting of uncoupled eNOS is highly beneficial in various animal models of cardiovascular and metabolic diseases [61]. Based on estimations, up to 1% of the electrons, which are involved in mitochondrial respiration, are transferred to molecular oxygen [62] O_2_^•^¯ is mainly formed at complex I as well as III [63] and potentially at complex II by reverse electron transport [64]. Mitochondria are a rich source of O_2_^•^¯, which is also supported by lethality of Mn-SOD deficiency [13,14], and mitochondrial O_2_^•^¯ formation plays a central role for ischemia/reperfusion damage (e.g., during myocardial infarction (MI) and stroke) [65,66]. Therefore, mitochondria-targeted antioxidants are currently investigated for a number of different diseases, including cardiovascular, neurodegenerative diseases, and diabetes [67,68,69]. MAO-A/B isoforms are flavoenzymes that catalyze the oxidative deamination of both endogenous and exogenous amines, including neurotransmitters and several drugs [70]. They are located at the outer mitochondrial membrane, produce H_2_O_2_ as a byproduct during normal enzymatic function, and have been investigated for therapy of neuronal disease and myocardial injury [56]. Upon activation and association with cytochrome c, p66^Shc^ represents an important source of H_2_O_2_ in myocardial ischemia/reperfusion but may also contribute to vascular abnormalities associated with diabetes and aging [71]. Of note, although genetic p66^Shc^ deletion mostly conferred antioxidant protective effects, it also plays a role in insulin signaling (which partly depends on a basal physiological reactive oxygen species (ROS) level) and is potentially involved in eustress [71], an endogenous protective preconditioning by mild oxidative stress as introduced above. It is well established that these RONS sources are also activated in the diabetic setting (see Section 3 for experimental and clinical evidence), suggesting that RONS contribute to diabetic cardiovascular and, potentially, other adverse health effects.

### 1.3. Preclinical/Molecular Proof of a Role of Oxidative Stress for Cardiometabolic Diseases

First evidence for a role of oxidative stress for cardiovascular complications in an experimental model of hypercholesterolemia is based on reports by Harrison and Ohara [72,73]. Later, genetic manipulations (e.g., knockout mice or transgenic overexpressing mice) provided a molecular proof of the involvement of ROS producing or detoxifying enzymes in the onset and progression of these cardiometabolic diseases (for review see [74]). We here just mention some prominent examples. Genetic deletion of the *p47^phox^* (a subunit of NOX2) improved vascular ^•^NO bioavailability in mice with MI, normalized ROS formation, and improved heart function (ejection fraction) as well as mortality after MI by 20% [75]. The cardiovascular complications in an AT-II-induced hypertension model, e.g., increased blood pressure and vascular ROS formation as well as impaired endothelial function, were largely absent in mice with *p47^phox^* or *Nox1* deficiency [76,77]. In contrast, transgenic mice overexpressing *Nox1* [78] and mice with deletion of antioxidant enzymes, e.g., by heterozygous *Mn-SOD* deficiency (*Sod2*^+/−^) [79,80], showed aggravated cardiovascular complications, especially with additional stress conditions. Deletion of the *Gpx1* in atherosclerosis-prone apolipoprotein E-deficient (*ApoE*^−/−^) mice caused accelerated atherosclerotic plaque formation and enhanced vascular oxidative stress [81], whereas *Gpx1* deficiency in wildtype mice resulted in more pronounced aging-associated complications [82]. Transgenic overexpression of GTP-cyclohydrolase-1, the enzyme that is responsible for de novo synthesis of BH4, is associated with improvement of cardiovascular complications in animal models of atherosclerosis [83], hypertension [84], and diabetes [85] by normalizing the coupling state and function of eNOS [86]. Genetic endothelial- or myelomonocytic-specific deficiency of the AMP-activated protein kinase (*AMPK*^fl/fl^Tekcre or *LysMcre* mice) resulted in endothelial dysfunction, vascular oxidative stress, and inflammation in AT-II-induced hypertension [87,88]. The contribution of oxidative stress to diabetic complications is substantial as demonstrated by the beneficial effects of antioxidant interventions, targeting mitochondrial O_2_^•^¯ formation in diabetic animals that largely prevented the adverse effects of hyperglycemia [89]. These data provide a molecular proof of the crucial role of oxidative stress in causing cardiovascular disease in animals (for review and more examples see [55,74,90,91,92]).

### 1.4. Clinical Evidence for a Role of Oxidative Stress in Cardiovascular and Metabolic Diseases

Oxidative stress is a hallmark of all cardiovascular diseases, confers activation of endothelial cells, and thereby facilitates adhesion/infiltration/activation of immune cells [55]. Oxidative stress is known to induce endothelial dysfunction [93] and to accelerate the progression of atherosclerosis [94]. The link between oxidative stress and cardiovascular prognosis is widely accepted and supported by data from large clinical trials. A positive correlation between levels of GPx-1 and cardiovascular event-free survival was reported by a large clinical study (636 subjects) [95]. Additionally, the serum levels of D-ROM (derivatives of reactive oxygen metabolites) that are indicative of ROS formation and the total thiol levels (representative of the total redox state) were strongly and independently associated with all-cause and cardiovascular mortality (10,622 subjects) [96]. The levels of 8-hydroxy-2-deoxyguanosine, a marker of oxidative DNA damage, were increased in patients with cardiovascular disease according to the data of a meta-analysis (1900 subjects) [97]. Additionally, a number of small cohort clinical studies support the concept that endothelial function (measured by flow-mediated dilation (FMD)), which represents a subclinical marker of atherosclerosis and early predictor for cardiovascular events [49,50], correlates with the oxidative stress burden as assessed by vitamin C responsiveness [93], reduced circulating glutathione levels [98], SOD activity or oxidized low-density lipoprotein (oxLDL, oxidative stress marker, and initiator of atherosclerosis), as well as malondialdehyde (MDA) or 8-oxo-deoxyguanosine levels [99]. A detailed review on the impact of oxidative stress on cardiovascular disease development and progression with a detailed list of clinical studies can be found in references [61,74,100,101].

The contribution of oxidative stress to the cardiovascular complications in diabetes is widely accepted [102,103]. A meta-analysis of 33 studies revealed that administration of vitamin D significantly reduced the serum levels of high-sensitivity C-reactive protein (hs-CRP) and MDA levels in diabetic patients, whereas vitamin D treatment increased ^•^NO bioavailability and the levels of reduced glutathione [104]. Although vitamin D is not a classical low-molecular-weight antioxidant compound, its beneficial effects on oxidative stress are more and more recognized [105,106,107,108]. A meta-analysis of 12 studies revealed that supplementation with vitamin E was associated with reduced blood glucose and glycated hemoglobin and administration of vitamins C as well as E were associated with lower MDA levels and higher GPx as well as SOD activity [109]. The ADVANCE Trial revealed that 8-oxo-2’-deoxyguanosine levels were correlated with all-cause and cardiovascular mortality in adults with type 2 diabetes mellitus (T2DM) (*n* = 3766) [110]. Likewise, an independent prospective cohort study revealed a correlation of RNA oxidation with all-cause and cardiovascular mortality risk in patients with T2DM [111]. Dapagliflozin therapy conferred better glycemic control, endothelial function, which was associated with lower 8-oxo-2’-deoxyguanosine urine levels in T2DM patients [112]. Oxidative degradation of BH4, the essential eNOS cofactor, was observed in diabetic patients and points to an uncoupled/dysfunctional eNOS, all of which was corrected by acute BH4 infusion [113]. In addition, treatment with the antioxidants lipoic acid or vitamin C normalized endothelial function in diabetic subjects [114]. A population-based study (631 subjects) revealed an association between T2DM and endothelial dysfunction as well as low-grade inflammation providing an explanation for a 43% higher cardiovascular mortality risk for diabetic subjects [115].

Despite the large body of evidence for a role of oxidative stress in disease development and progression, almost all large clinical trials for nonselective antioxidant therapy (mainly vitamins C and E, chronic high dose oral administration) failed to show any health benefit for the treatment of cardiovascular disease [12,116]. Only a few exceptions are published such as the European Prospective Investigation into Cancer (EPIC)-Norfolk study with measurement of vitamin C plasma levels of all participants [117] and small-cohort studies using acute high-dose administration (mainly infusion) of vitamins (reviewed in [49,118]). Additionally, the quite expensive development of the synthetic antioxidant NXY-059 is not an exception and failed to prove benefits in clinical studies with stroke patients [119]. The most likely explanations for the rather disappointing outcome of clinical antioxidant trials so far, previously discussed in very detail but comprise among others, were the limited up-take of classic oral antioxidants in tissues undergoing oxidative stress, access to intracellular sites of ROS production, the limited reactivity towards specific ROS (e.g., H_2_O_2_ or O_2_^•^¯), and most importantly, interference with essential physiological ROS signaling [12,49,91].

## 2. Cross talk between Different Sources of RONS

### 2.1. Interaction of Different RONS Sources—the Concept of “ROS-Triggered ROS Formation”

**Figure 3:**
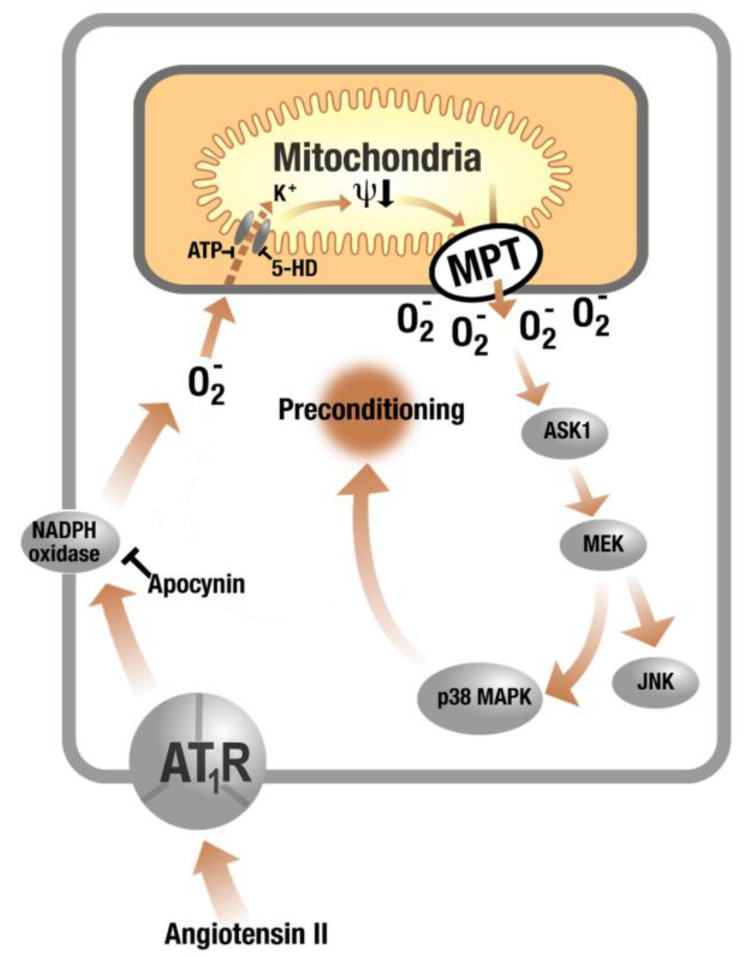
Cross talk of NOX and mitochondria in AT-II-triggered ROS formation. AT-II-triggered activation of NOX in the cytoplasmic membrane with subsequent opening of mitochondrial K_ATP_ channels by NOX-derived ROS and mitochondrial ROS release via mPTP opening (in the figure termed MPT). Upon mtK_ATP_ opening, the electrophoretic influx of potassium cations into the matrix causes depolarization of the mitochondrial membrane (Δψ_m_ ↓) along with matrix swelling and alkalinization [131]. Matrix alkalinization, in turn, has been suggested to be responsible for the increase in H_2_O_2_ formation observed in cardiomyocytes treated with the mtK_ATP_ opener diazoxide [132,133,134]. The entire process was blocked by the specific mtK_ATP_ inhibitor 5-hydroxydecanoate (5-HD). This concept provides an amplification mechanism for AT-II-induced oxidative stress and contribute to AT-II-mediated preconditioning via P38 mitogen-activated protein kinases (p38 MAPK) and c-Jun N-terminal kinase (JNK) pathway. AT_1_R, angiotensin II type 1 receptor. Modified from [123] and adapted from [92] with permission.

**Figure 4:**
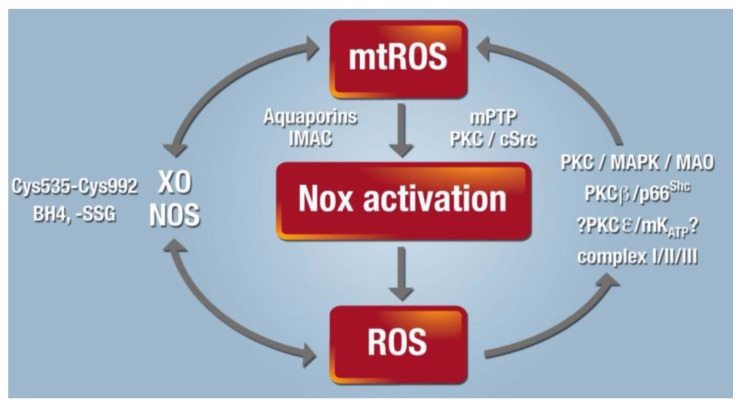
Cross talk between different sources of RONS (mitochondria, NOX, XO, and uncoupled NOS). XO originates from oxidative stress-mediated conversion of the xanthine dehydrogenase via oxidation of critical thiols in cysteine535/992 [143,144]. NOS (mainly eNOS) are uncoupled upon oxidative depletion of BH4 [129], *S*-glutathionylation (-SSG) [145], adverse phosphorylation by PKC [146] or protein tyrosine kinase-2 [147], and other redox switches (reviewed in [90]). Mitochondrial O_2_^•^¯/H_2_O_2_ formation is triggered by oxidative stress from all ROS sources (including other damaged/activated mitochondria) via redox-activation of PKC, mitogen-activated protein kinases (MAPK), other kinase pathways, and potential involvement of redox-sensitive mtK_ATP_ with subsequent p66^Shc^, MAO, respiratory complex activation, or impairment of mitochondrial antioxidant defense (reviewed in [127]). Mitochondrial O_2_^•^¯/H_2_O_2_ is released to the cytosol via mitochondrial pores and channels (e.g., redox-sensitive mPTP, inner membrane anion channel (IMAC) or aquaporins) or by diffusion due to increased mitochondrial permeability under pro-inflammatory conditions (reviewed in [127]). In the cytosol, these species (along with released calcium) cause activation of redox-sensitive PKC and tyrosine kinases (cSrc) with subsequent activation of NOX and amplification of the cellular oxidative stress [139]. Adapted from [127] with permission.

The concept of “ROS-triggered ROS formation” was first reported for self-amplified mitochondrial ROS formation envisaged by waves of enhanced ROS levels along mitochondrial networks (dysfunctional mitochondria release ROS that stimulate ROS release by neighbored mitochondria) [120]. This concept was extensively reviewed with all mechanistic details by Zorov and colleagues [121]. Later, this concept was extended not only to interaction of NOX and mitochondria in AT-II-mediated preconditioning [122] as well as adverse effects [64] but also to other disease settings and redox processes [92]. Kimura et al. established that AT-II-stimulated NOX-dependent ROS formation in the myocardium confers ischemic preconditioning [122]. These protective effects were blocked by the NOX inhibitor apocynin and blockade of the mitochondrial ATP-sensitive potassium channels (mtK_ATP_) in cardiac myocytes by 5-hydroxydecanoate (5-HD). In an editorial to this original paper, Brandes proposed that cytosolic ROS generated by NOX can stimulate mitochondrial ROS formation [123]. The mechanism could be based on activation of mtK_ATP_ in the mitochondrial membrane by NOX-derived cytosolic ROS with subsequent opening of the permeability transition pore (mPTP, in the figure termed MPT) (Figure 3) [124]. In general, the concept of “kindling radicals” (or also “bonfire” hypothesis) is known for long time [125] and provides an attractive explanation for the activation of secondary ROS sources and functional damage of redox-regulated enzymes such as eNOS [55,126]. In summary, initial formation of ROS (most likely from NADPH oxidases or mitochondria) leads to further oxidative damage of key enzymes such as eNOS via different uncoupling mechanisms (see “redox switches” in Figure 4) [127,128]. The ROS-induced ROS production concept can be extended to almost any kind of source of RONS as almost all of these sources contain “redox switches.” For hypertension, it has been repeatedly shown that genetic deficiency in NADPH oxidase subunits, especially knockout of the phagocytic isoform NOX2 eliminating the superoxide formation from phagocytes, prevents eNOS uncoupling and endothelial dysfunction [129]. Similar observations on eNOS uncoupling were made in cultured endothelial cells upon challenges with typical biological oxidants such as peroxynitrite or hypochlorous acid as well as in Nox2^−/−^ mice [130]. Accordingly, NOX2 is a likely candidate for the generation of the “kindling radicals.”

The concept of the interaction (cross talk) of different ROS sources was developed to explain the observation that pharmacological inhibition or genetic deletion of one specific ROS source is in many disease models enough to confer a complete normalization of the disease phenotype (see numerous examples for hypertension and myocardial infarction (MI) in reference [127]). The reports on a complete normalization of hypertensive complications (including higher cardiovascular ROS formation) upon genetic deletion of *Nox1, Nox2*, or *Nox4* as well as pharmacological inhibition of mitochondrial or XO-derived ROS either mean that some of these reports are not correct or that all ROS sources interact in a cross talk fashion and activate each other, with the logical conclusion that inhibition of only one of these sources is enough to prevent oxidative damage and normalize the overall ROS formation [127]. However, the cross talk between different ROS sources was also suggested to play a role in vascular cellular redox signaling, especially when substantial local ROS accumulation is required for redox-triggered processes [135]. The mechanism behind this concept is that each ROS source has so-called “redox switches” that confer activation upon oxidation [90,92,136] (Figure 4). Although this redox cross talk was initially demonstrated for the NOX2/mitochondrial axis in the setting of hypertension [137,138,139], nitrate tolerance [140], and aging [79,82,139], it can be extended to other ROS-producing enzymes such as uncoupled eNOS [90,139] and xanthine dehydrogenase/oxidase conversion [64,90] (Figure 4) as well as to other disease settings. Especially, the role of cyclophilin D (CypD), a small redox-sensitive regulator of the mPTP, in the cross talk of mitochondrial ROS and NOX2-dependent ROS formation is meanwhile well established in AT-II-induced hypertension, by prevention of most adverse effects in *CypD* knockout mice [139,141]. Cysteine 203 in CypD determines the activity of the mPTP regulator CypD, and therefore, represents a redox switch of mPTP which confers higher opening probability of the pore under oxidative stress conditions [142]. In contrast, S-nitros(yl)ation of cysteine 203 prevented H_2_O_2_-induced mPTP opening identifying nitric oxide as an antagonist of ROS in this redox process.

Ischemia/reperfusion damage is based on mitochondrial ROS formation as a central pathophysiological mechanism [148,149,150]. Rathore et al. reported a mechanism by which mitochondrial ROS activate PKCε (prevented by chelerythrine and *PKCε* deletion) with subsequent increase in NOX activity (prevented by apocynin and *p47^phox^* deletion) in the setting of hypoxia as a model of ischemia/reperfusion damage (e.g., as observed in myocardial infarction (MI) or stroke) [151]. The authors show that hypoxia activates most likely NOX1 isoform in pulmonary arteries as documented by translocation of p47^phox^ to the plasma membrane. The involvement of mitochondrial ROS formation in this process was proven by lower NADPH activity in *Gpx1* overexpressing mice and higher NADPH activity in *Gpx1* knockout mice. A cross talk between mitochondria and NOX1 or NOX2 was also shown for cellular starvation [152], nitrate tolerance [140], the aging process [79,82,139,153], AGE/RAGE signaling [154], endotoxemia as a model of sepsis [155], dyspneic patients with uremic lung injury [156], AsO_3_ toxicity [157], idiopathic pulmonary fibrosis [158], and tumorigenesis [159]. Even a triple cross talk between NOX4, NOX2, and mitochondria as well as ROS-induced ROS release was described in vascular endothelial growth factor (VEGF) signaling and angiogenesis [160]. Oxidative stress in general and this cross talk in particular have also large impact on cellular calcium homeostasis and mitochondrial function in the diabetic heart [161], similar to the Ca^2+^/ROS cross talk previously described in cancer development and progression [162] and cellular function per se [163]. Of note, similar cross talk mechanisms are likely in the setting of diabetes as all major RONS sources are activated under hyperglycemic conditions as reviewed in [92,127] and discussed in detail in Section 3 of the present work.

### 2.2. Cross Talk of Oxidative Stress and Inflammation

The link between vascular dysfunction and cardiovascular diseases such as arterial hypertension, hypercholesterolemia, and coronary artery disease can be best explained by inflammation [100,128]. Recent data support this tight association between redox regulatory pathways and inflammation via redox activation of immune cells by mitochondrial O_2_^•^¯/H_2_O_2_ and the subsequent activation of the phagocytic NOX2 [139,164]. NOX2 is efficiently activated by mitochondrial O_2_^•^¯/H_2_O_2_ formation via the before described redox cross talk [90,127], a process that is key to the activation, recruitment, and infiltration of myelomonocytic cells [165,166] and T cells [167]. Likewise, blood pressure in hypertensive humanized mice was normalized when infiltration of immune cells was prevented [168], supporting the concept that inflammatory processes and NOX2 in immune cells are driving vascular dysfunction. This assumption is in accordance with previous observations that the cellular redox state controls the activity and inflammatory potential of macrophages [169,170]. Mitochondrial ROS formation can cause opening of the mPTP, which chronically causes disruption of mitochondria with subsequent unspecific release of (oxidized) mtDNA, a damage-associated molecular pattern (DAMP), leading to “sterile inflammation” [128,171]. Other examples for the redox regulation of inflammatory pathways are redox modifications of mediators of inflammation (e.g., high-mobility group protein 1 (HMGB1), S100 proteins, and damage-associated molecular patterns (DAMPs)) and modulation of transcription factors related to inflammation (e.g., nuclear factor erythroid 2-related factor 2 (NRF2), activator protein 1 (AP-1), nuclear factor kappa-light-chain-enhancer of activated B cells (NF-κB), and hypoxia-inducible factor 1-alpha (HIF-1α)) [128]. A molecular basis for this cross talk between oxidative stress and inflammation was provided by identification of substantial redox regulation of the NLR Family Pyrin Domain Containing 3 (NLRP3) inflammasome that controls the release of cytokines [172,173,174,175,176,177], the central organizer of inflammation high-mobility group box 1 (HMGB1) [178,179,180,181,182] and the antibacterial process of neutrophil extracellular traps (NETs) formation [183,184,185,186,187,188] as well as other processes reviewed in [128]. Another molecular proof of the large impact of oxidative stress on inflammation comes from animal models with genetic knockout of antioxidant defense enzymes, all of which displaying an inflammatory phenotype [100]. The link between (mitochondrial) ROS formation and inflammation [189] also disproved the previous opinion that ROS produced in the mitochondria are only unwanted by-products of oxidative metabolism and put-forward the concept that mitochondrial ROS represent a nexus of cellular homeostasis [184]. An aggravated cross talk between oxidative stress and inflammation may also be expected in the setting of diabetes as oxidative stress is increased and inflammatory markers are upregulated under hyperglycemic conditions as reviewed in [103].

### 2.3. Glucotoxicity and AGE/RAGE Signaling

Hyperglycemia causes modifications of proteins by nonenzymatic glycosylation, leading to the formation of advanced glycation end products (AGEs) [190], which contribute to endothelial dysfunction [191]. AGE/RAGE signaling in diabetic rats also triggers vascular complications via NOX-induced oxidative stress [192], mitochondrial ROS formation [154], and inflammation with atherosclerosis [193]. Macrophages from *gp91^phox^* (*Nox2*) null mice responded less efficiently to AGE stimulation, whereas cultured endothelial cells showed inflammatory activation by AGE envisaged by vascular cell adhesion molecule 1 (VCAM-1) upregulation [192]. Multiple-antioxidant therapy prevented higher expression of tumor necrosis factor alpha (TNF-α), interferon gamma (IFN-γ), and ROS-producing enzymes in a type 1 diabetes mellitus (T1DM) model [194], clearly supporting an association of oxidative stress, inflammation, and diabetic complications. Cardiovascular complications of diabetes are most probably initiated by AGE/RAGE signaling and diacylglycerol (DAG) formation due to higher AT-II levels (reviewed in [195]). Besides these mechanisms, enhanced PKC activity, hexosamine metabolism, and sorbitol production by the polyol pathway most likely contribute to the diabetic phenotype, which is also characterized by increased expression of inflammatory cytokines and plasminogen activator inhibitor-1 (PAI-1) [196]. We established correlations between oxidative stress, AGE/RAGE signaling, inflammation, and endothelial function in a model of T2DM (ZDF rats) with empagliflozin (SGLT2 inhibitor) therapy [197], pointing towards vital cross talk between these parameters.

### 2.4. Other Redox Switches—Link between Oxidative stress, Inflammation, and Vascular Function

The endogenous eNOS inhibitor asymmetric dimethyl arginine (ADMA) activates neutrophils and release of myeloperoxidase (MPO) leading to inhibition of dimethylaminohydrolases (DDAH), the enzyme catalyzing the breakdown of asymmetric dimethyl arginine (ADMA), and endothelial dysfunction [198]. Inhibition of sGC leads to decreased cGMP levels and loss of its anti-inflammatory effects [199,200]. Overall, the redox control of transcription factors of central importance represents a global redox switch that can affect almost all cellular pathways [201]. The sympathetic nervous system is activated under oxidative stress conditions leading to the release of vasoconstrictors (e.g., catecholamines) as demonstrated in different models of hypertension [202,203,204]. Likewise, the renin–angiotensin–aldosterone system (RAAS) is activated by ROS formation in different models of hypertension [165,205,206,207] and endothelin-1 generation by oxidative stabilization of its promoter [208]. Prostacyclin synthase (PGIS) is oxidatively inhibited via nitration of tyrosine 430 by ONOO¯, whereas peroxide-driven activation of cyclooxygenase-1/2 (COX-1/2) leads to higher prostaglandin endoperoxide (PGH2) levels and cyclooxygenase-2 (COX-2) is oxidatively inhibited via tyrosine nitration, all of which contributes to redox regulation of prostanoid synthesis and vascular tone with relevance for atherosclerosis, diabetes, nitrate tolerance, and sepsis [8,49,209,210,211,212,213,214,215]. Additionally, sGC is redox regulated via thiol oxidation and S-nitros(yl)ation, whereas oxidative activation and upregulation of phosphodiesterases lead to enhanced break-down of cGMP, all of which contribute to modulation of the NO/cGMP signaling cascade [47,49,216,217,218,219,220]. There are many prominent examples of well-established S-nitrosation-regulated enzymes such as caspase activity and initiation of apoptosis [221] as well as other examples reviewed elsewhere [222,223]. S-nitrosation per se is highly redox regulated as it requires the interaction of ^•^NO and O_2_^•^¯ [224], and the optimal nitrosative conditions require tight control of the ^•^NO/O_2_^•^¯ ratio (should be 3:1) to generate the potent nitrosating species N_2_O_3_ [225]. However, some evidence suggests that S-nitros(yl)ation plays not a central role in redox regulation [226]. There are multiple other redox switches in the cardiovascular system regulating the glycocalyx, thrombosis and coagulation, inflammation, vasoconstrictors such as endothelin-1, fibrosis, calcification, and smooth muscle cell proliferation that are all important for cardiovascular health and disease. It would be beyond the scope of this review to list them all since they are summarized in a previous review [74]. Importantly, many of these alternative redox switches are dysregulated in the setting of diabetes as exemplified by enhanced degradation of the glycocalyx [227], activation of the RAAS as supported by successful medication of diabetic patients with cardiovascular complications with angiotensin-converting enzyme inhibitors or AT1-receptor blockers [228,229], and higher endothelin-1 plasma levels in diabetic patients [230].

## 3. Evidence for a Cross Talk between Different Sources of ROS in the Setting of Diabetes

**Figure 5:**
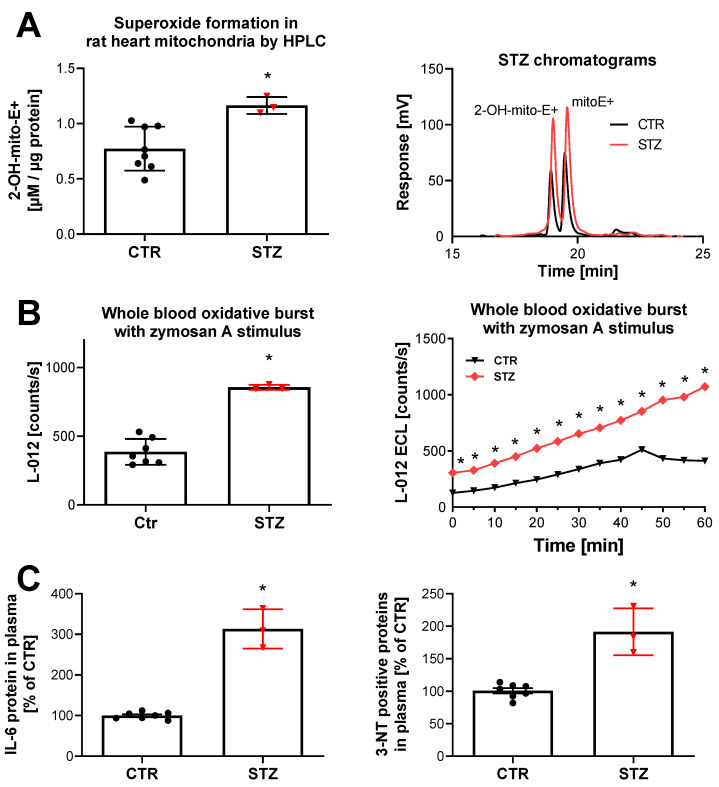
Detection of mitochondria and NOX-derived ROS formation as well as inflammation and nitro-oxidative stress markers in T1DM rats. (**A**) The yield of the superoxide-specific mitoSOX oxidation product triphenylphosphonium 2-hydroxy ethidium (2-OH-mito-E+) in mitochondrial preparations of diabetic (STZ) and respective control animals. Representative chromatograms are shown for the HPLC-based quantification of 2-OH-mito-E+. (**B**) Detection of ROS formation during oxidative burst in whole blood from diabetic rats. Quantification of ROS formation by L-012 (100 µM) ECL in response to stimulus by zymosan A. Representative kinetic traces are shown for 1 animal per group upon zymosan A (Ctr vs. STZ) stimulation with four technical replicates per data point. (**C**) Biomarkers of inflammation (IL-6) and oxidative stress by tyrosine nitration (3-NT) were increased in plasma of diabetic rats as compared to control animals. Each data point in the bar graphs represents one animal. * means *p* < 0.05 versus control. Adapted from [236] with permission under the Creative Commons Attribution License agreement.

There is evidence for activation of multiple ROS sources in the setting of diabetes. Increased NOX1 but not NOX4 expression in the aorta of T1DM rats (streptozotocin (STZ) model) go hand in hand with enhanced eNOS uncoupling, XO activity, and mitochondrial ROS formation [231]. Cardiac NOX and serum XO activity are increased and NOX1/2 expression as well as eNOS uncoupling (measured by S-glutathionylation) are enhanced in T1DM rats (STZ model), which was partially normalized by treatment with an antioxidant organic nitrate (pentaerithrityl tetranitrate, PETN) via nuclear factor erythroid 2-related factor 2 (NRF2) activation [232]. Histone deacetylases HDAC1/2 interact with promoters of *Nox* isoforms and play a role for NOX upregulation in experimental diabetes, which was prevented by a pan-HDAC inhibitor and aggravated by histone deacetylases 2 (HDAC2) overexpression [233]. NOX activity and expression of subunits p22^phox^, p67^phox^, and p47^phox^ were increased in bypass vascular tissues of diabetic patients [234]. T2DM rats display higher NOX activity in leukocytes with increasing hyperglycemia (higher glycated hemoglobin (HbA1c) levels), which was associated with more pronounced mitochondrial oxidative stress (decreased aldehyde dehydrogenase 2 (ALDH-2) activity) and systemic inflammation as well as AGE/RAGE signaling (markers such as C-reactive protein (CRP) and methylglyoxal), all of which was normalized by glucosuria therapy (sodium/glucose cotransporter 2 (SGLT2) inhibition) [197]. Similar observations were also made in a T1DM with sodium/glucose cotransporter 2 (SGLT2) inhibition [235]. Enhanced mitochondrial O_2_^•^¯ formation, NADPH oxidase activity in leukocytes (oxidative burst), and inflammatory markers interleukin-6 as well as 3-nitrotyrosine (probably from inducible NOS (iNOS) and ONOO¯ formation) were also recently reported (Figure 5) [236].

T1DM is associated with enhanced *Nox1* and *Nox2* expression and activity as well as eNOS uncoupling, all of which was prevented by angiotensin-1 receptor blockade [237]. In accordance to our cross talk concept in the setting of diabetes, NADPH oxidases produce the “kindling radicals” leading to uncoupling of eNOS via the above described “redox switches” (e.g., BH4 depletion and S-glutathionylation) and may also contribute to direct dysfunction of eNOS by PKC-dependent phosphorylation of eNOS at Thr495 [55]. Albuminuria, kidney damage, and other major diabetic complications are initiated by NOX- and mitochondria-derived ROS formation with adverse signaling of down-stream kinases, caspases, and redox-sensitive transcription factors [238]. Initial evidence for a cross talk between different sources of ROS in the setting of diabetes comes from the observation that diabetic complications are prevented by specific inhibitors of single ROS sources (Table 1). Unfortunately, targeting the mPTP by *CypD* knockout in the setting of diabetes did not prevent diabetic renal damage [239], representing a drawback for the hypothesis that we have put forward above. Finally, also a cross talk between oxidative stress and inflammation may be expected in the setting of diabetes, as markers of inflammation are increased in diabetic patients [240,241]. Markers of inflammation and oxidative stress were also substantially decreased by SGLT2 inhibitor therapy in T1DM and T2DM animal models [197,235]. This may be also related to the fact that inflammation and oxidative stress are interconnected by AGE/RAGE signaling [100].

## 4. Conclusions

**Figure 6:**
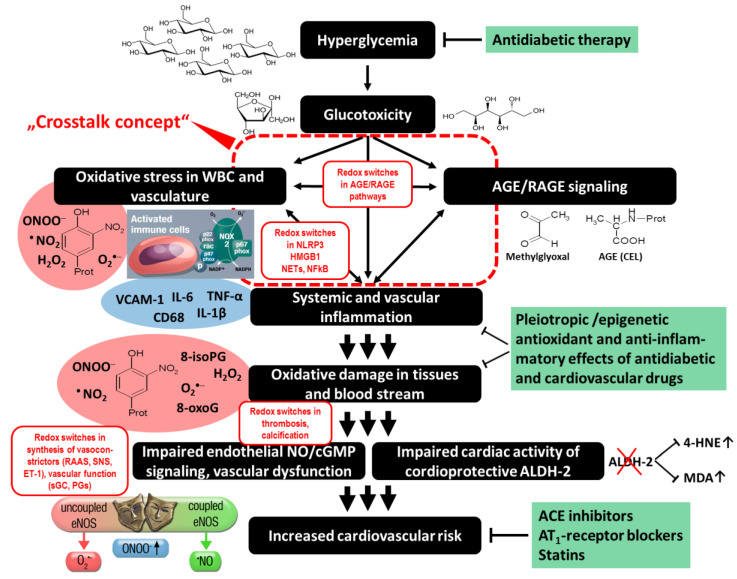
Extension of the cross talk concept from ROS sources to pathways initiated by hyperglycemia and glucotoxicity (fructose and sorbitol overproduction) such as inflammation (typical diabetic markers in the blue elliptic box), AGE/RAGE signaling (typical AGE members: precursor methylglyoxal and protein adduct N^ε^-(carboxylethyl)-l-lysine), synthesis of vasoconstrictors, regulation of thrombosis, calcification, and vascular function. Typical diabetic ROS and oxidative damage markers are shown in the red elliptic boxes. The major therapeutic targets of current antidiabetic and cardiovascular therapies are reflected by green text boxes. NETs, neutrophil extracellular traps; NLRP3, NLR Family Pyrin Domain Containing 3 inflammasome; HMGB1, high-mobility group box 1; VCAM-1, vascular cell adhesion molecule-1; IL, interleukin; TNF-α, tumor necrosis factor alpha; CD68, cluster of differentiation 68 (macrosialin); 8-isoPG, 8-isoprostane; 8-oxoG, 8-oxoguanine; RAAS, renin–angiotensin–aldosterone system; SNS, sympathetic nervous system; ET-1, endothelin-1; mtROS, mitochondrial ROS; DAMPs, damage-associated molecular patterns; PGs, prostaglandins; ALDH-2, mitochondrial aldehyde dehydrogenase; 4-HNE, 4-hydroxynonenal; MDA, malondialdehyde; ACE, angiotensin-converting enzyme; AT_1_-receptor, angiotensin II type 1 receptor. Significantly modified from [197] under the terms and conditions of the Creative Commons Attribution License.

With the present review, we want to highlight redox signaling in physiology and disease emphasizing the cross talk of different ROS sources. NOX and mitochondria obviously represent a redox tandem playing a central role in many diseases [92,127]. Redox signaling may become an attractive target for drug development in the future, but its complexity warrants in-depth mechanistic understanding and careful fine-tuning since ROS not only are by-products causing damage but also fulfill essential physiological signaling functions [11]. Especially, the fact that this cross talk is not limited to the interplay of different ROS sources but can be extended to interactions of ROS with inflammatory pathways, AGE/RAGE signaling, vasoconstrictor synthesis, thrombosis/coagulation, and very clearly endothelial function (Figure 6), makes therapeutic targeting complicated. Mitochondria-targeted antioxidants and specific NOX isoform inhibitors constitute promising present and future approaches. Control of mitochondrial channels such as the mPTP or the mtK_ATP_ seems to be an attractive therapeutic strategy [65,131], as also considered for treatment of brain disorders [261] and combatting diabetic complications [262]. In addition, the interplay of (mitochondrial) ROS and the NLRP3 inflammasome represents an attractive therapeutic target that needs to be investigated for exploitation in more detail [189]. A number of antioxidant treatments was suggested for the mitochondria/NOX cross talk underlying the complications of idiopathic pulmonary fibrosis, but further evaluation by translational approaches is necessary [158]. Of note, also interfering with the redox cross talk between mitochondria and NOX most likely requires careful fine-tuning since ROS-induced ROS release via this cross talk is also implicated in physiological processes such as flow-mediated dilation (FMD) of microvessels [263].

## Figures and Tables

**Table 1:** Contribution of different ROS sources to the severity of diabetic complications.

Studies and Major Outcomes	Ref.
T1DM rats (STZ model) show sevenfold increase in *gp91^phox^* (*Nox2*) mRNA and uncoupled eNOS—diabetic complications were partially normalized by inhibition of PKC by chelerythrine	[242]
Genetic deletion of *NoxO1* or *p47^phox^* reduced blood pressure and prevented diabetes-induced vascular dysfunction	[243]
Combined NOX1/4 inhibition with GKT137831 in mice provides dose-dependent reno- and atheroprotection even in established micro- and macrovascular disease	[244]
*Nox1* deficiency normalized diabetic glomerular DNA damage	[245]
NOX1 plays a key role in diabetes mellitus-accelerated atherosclerosis, which can be prevented by siRNA against *Nox1* and GKT137831 therapy	[246]
Critical role for NOX2 in insulin resistance-related endothelial cell dysfunction as demonstrated by genetic deletion of *Nox2*	[247]
*Nox2* deficiency protects against STZ-induced beta-cell destruction and development of diabetes in mice	[248]
Normalization of mitochondrial ROS formation prevents several diabetic complications (glucose-induced activation of protein kinase C, formation of AGEs, sorbitol accumulation, and NFkB activation)	[89]
Blocking mitochondrial ROS formation with mitoTEMPO prevented diabetic cardiomyopathy	[249]
The mitochondria-targeted antioxidant MitoQ ameliorated tubular injury mediated by mitophagy in diabetic kidney disease via NRF2/PINK1	[250]
Deletion of *p66^Shc^* longevity gene protects against experimental diabetic glomerulopathy by preventing diabetes-induced oxidative stress	[251]
Diabetes promotes cardiac stem cell aging and heart failure, which are prevented by deletion of the *p66^Shc^* gene	[252]
Mammalian life-span determinant p66^Shc^A mediates obesity-induced insulin resistance	[253]
MAO-dependent endoplasmic reticulum-mitochondria dysfunction and mast cell degranulation lead to adverse cardiac remodeling in diabetes, which was supported by protective effects of MAO inhibito rpargyline	[254]
Emerging role of MAO as a therapeutic target for cardiovascular disease including treatment of diabetes	[255]
XO is activated in human and experimental T1DM, and the XO inhibitor allopurinol normalizes major diabetic complications	[256]
Atrial remodeling was prevented by allopurinol in diabetic rabbits (alloxan model)	[257]
XO inhibitor febuxostat exerts an anti-inflammatory action and protects against diabetic nephropathy development in KK-Ay obese diabetic mice	[258]
*eNOS* gene therapy exacerbates hepatic ischemia-reperfusion injury in diabetes, which was normalized by BH4 or the BH4 precursor sepiapterin providing evidence for eNOS uncoupling	[259]
Oxidation of the zinc–thiolate complex and uncoupling of eNOS by ONOO¯ as an explanation for endothelial dysfunction in the setting of diabetes	[260]
Overexpression of the BH4-generating enzyme GTP-cyclohydrolase-1 rescues eNOS function in diabetic mice indicating oxidative BH4 depletion in this model	[85]

## Abbreviations

AGEs	Advanced glycation end products
AT-II	Angiotensin II
BH4	Tetrahydrobiopterin
CRP	C-reactive protein
CypD	Cyclophilin D
EDRF	Endothelium-derived relaxing factor
eNOS	Endothelial nitric oxide synthase
GPx	Glutathione peroxidases
H_2_O_2_	Hydrogen peroxide
HO^•^	Hydroxyl radical
MDA	Malondialdehyde
MAO	Monoamine oxidase
MI	Myocardial infarction
Mn-SOD	Mitochondrial superoxide dismutase
mPTP	Mitochondrial permeability transition pore
mtK_ATP_ NOX	Mitochondrial ATP-sensitive potassium channels
^•^NO	NADPH oxidases Nitric oxide
NOS	Nitric oxide synthase
NLRP3	NLR Family Pyrin Domain Containing 3
O_2_^•^¯	Superoxide anion radical, superoxide
PKC	protein kinase C
RONS	Reactive oxygen and nitrogen species
ROS	Reactive oxygen species
sGC	Soluble guanylyl cyclase
SOD	Superoxide dismutase
STZ	Streptozotocin
T1DM	Type 1 diabetes mellitus
T2DM	Type 2 diabetes mellitus
ucNOS	Uncoupled nitric oxide synthase
XO	Xanthine oxidase
